# Dynamic Reconstruction and Microenvironment Modulation of a Pd-Doped CuS Electrocatalyst for Nearly Unity-Efficiency Ammonia Electrosynthesis from Nitrate

**DOI:** 10.1021/jacs.5c16232

**Published:** 2025-11-06

**Authors:** Qun He, Zhangsheng Shi, Dongxue Yu, Yunpeng Zuo, Wei Jiang, Hengjie Liu, Chuanqiang Wu, Li Song, Xin Wang

**Affiliations:** † Department of Chemistry, 53025 City University of Hong Kong, Kowloon 999077, China; ‡ National Synchrotron Radiation Laboratory, 12652 University of Science and Technology of China, Hefei 230029, China; § Information Materials and Intelligent Sensing Laboratory of Anhui Province, Key Laboratory of Structure and Functional Regulation of Hybrid Materials of Ministry of Education, Institutes of Physical Science and Information Technology, 12487 Anhui University, Hefei 230601, China

## Abstract

Copper-based catalysts are known for their high ammonia selectivity in the electrochemical nitrate reduction reaction (NO_3_RR), yet the underlying mechanisms of this selectivity remain insufficiently understood and warrant further investigation. This study employs single-atom palladium-doped copper sulfide (Pd_1_/CuS) as a model precatalyst to elucidate the mechanisms driving its high selectivity. Comprehensive characterization reveals that Pd_1_/CuS undergoes in situ transformation into Cu while maintaining isolated palladium sites. The activated catalyst achieves near-unity (∼100%) selectivity for ammonia at −0.5 V vs RHE, along with high yield rates that significantly surpass those of the undoped catalyst, maintaining over 98.2% selectivity across 15 consecutive cycles. Mechanistic studies using in situ spectroscopies, theoretical calculations, and ab initio molecular dynamics simulations demonstrate that the incorporation of Pd promotes the partial desolvation of hydrated alkali ions, enhances water dissociation, improves intermediate adsorption in NO_3_RR, and facilitates proton transfer by strengthening the hydrogen-bond network while thermodynamically suppressing the recombination of adsorbed protons (*H). These effects synergistically promote the ammonia-selective pathway. This work provides fundamental insights into the relationship between dynamic structural evolution and catalytic performance in the NO_3_RR, advancing the rational design of high-selectivity copper-based catalysts.

## Graphical abstract

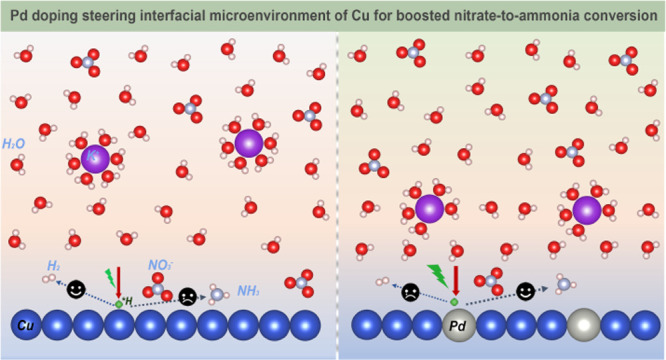

## Introduction

Nitrate pollution presents a global environmental challenge, as its excessive accumulation not only eutrophicates water bodies but also biologically transforms into carcinogenic nitrites, posing significant threats to ecosystems and human health.^1,2^ Traditional nitrate treatment technologies, including biological denitrification and ion exchange, are inefficient, energy-intensive, and produce secondary pollution, failing to meet the requirements of sustainable development.^3,4^ Recently, electrochemical nitrate reduction reaction (NO_3_RR) has garnered attention, as it can convert nitrates into harmless nitrogen or valuable ammonia (NH_3_) under mild conditions and can be integrated with renewable energy sources.^5–8^ NH_3_, an important chemical raw material and a potential future clean energy carrier, can be synthesized through this process, contributing to the carbon neutrality goals. However, the industrially dominant Haber–Bosch process requires high temperature and pressure, leading to significant energy consumption and carbon emissions.^9^ This led researchers to explore electrocatalytic nitrate reduction as an alternative pathway.

In the realm of electrocatalytic NO_3_RR, catalysts based on copper (Cu) display promising application potential, owing to their strong nitrate adsorption capabilities and moderate hydrogen adsorption energy.^10–13^ For instance, Cu nanowires have been shown to achieve an NH_3_ Faradaic efficiency (FE) of 96.6% at −0.5 V vs the reversible hydrogen electrode (RHE).^14^ However, the competing hydrogen evolution reaction (HER) becomes more pronounced at elevated overpotentials. To enhance selectivity, researchers have refined the electronic structure of Cu using various approaches such as alloying (e.g., with Sn, Ru, Au, Pd) and defect engineering (e.g., introducing vacancies).^15–20^ These methodologies markedly boost the catalytic performance largely due to the emergence of synergistic effects. For example, a catalyst comprising reduced-graphene-oxide-supported RuCu alloy (Ru_1_Cu_10_/rGO) employed for the direct reduction of NO_3_^–^ to NH_3_ can achieve an NH_3_ formation rate of 0.38 mmol cm^–2^ h^–1^ and an FE of 98% for NH_3_.^21^ The impressive efficiency of Ru_1_Cu_10_/rGO can be attributed to the synergistic effect between Ru and Cu sites via a relay catalysis process. In this process, Cu demonstrates exclusive efficiency for the reduction of NO_3_^–^ to nitrite (NO_2_^–^), while Ru displays superior activity for the reduction of NO_2_^–^ to NH_3_. Moreover, the introduction of Ru into Cu modifies the d-band center of the alloy and effectively regulates the adsorption energy of NO_3_^–^ and NO_2_^–^, thereby enhancing the direct reduction of NO_3_^–^ to NH_3_. Although numerous catalysts have been employed for NO_3_RR, a significant challenge in mechanism research is the lack of a comprehensive understanding of the dynamic structural evolution process driven by electrochemistry in existing studies.^22–29^ This includes the state of real active sites and their impact mechanism on the interfacial microenvironment, which remain unclear.^30^ Addressing these key scientific issues is essential for a thorough understanding of the catalytic mechanism and for guiding future catalyst design.

In this study, we selected single-atom Pd-doped CuS (Pd_1_/CuS) as a model precatalyst to systematically probe the dynamic structural evolution and interfacial microenvironment modulation during NO_3_RR. The choice of CuS as a host is motivated by its well-defined crystalline structure and propensity for electrochemical transformation, which allows clear tracking of reconstruction processes. The introduction of Pd was chosen based on its suitable electronic structure and hydrogen affinity. Using a combination of multispectroscopic characterization techniques and electron microscopy, we elucidated the structural evolution of the precatalysts into a metallic phase under operating conditions, while the isolated palladium sites remained. The isolated Pd sites serve to suppress unintended hydrogen recombination on contiguous Pd–Pd sites. This unique configuration further establishes a precision-tuned active microenvironment via highly localized Pd–Cu dual sites, promoting a higher ammonia selectivity. Further in situ spectroscopic analyses, theoretical calculations, and ab initio molecular dynamics simulations (AIMD) revealed that the incorporated Pd sites disfavored the HER by increasing the energy barrier for the recombination of adsorbed protons (*H); simultaneously, the Volmer step (water dissociation) was promoted, enhancing proton supply, while the strengthened interfacial hydrogen-bond network facilitated *H diffusion, thereby boosting hydrogenation processes. These synergistic effects endowed the Pd-doped catalyst with exceptional NO_3_RR performance, achieving a near-unity FE of ammonia (∼100%) at −0.5 V vs RHE and an impressive ammonia yield rate of ∼4350 mmol h^–1^ g_cat_^–1^, significantly outperforming the undoped catalyst. Moreover, the catalyst demonstrated excellent stability, maintaining a greater than 98.2% ammonia selectivity across 15 consecutive electrolysis cycles.

## Results and Discussion

### Structural Analysis of Catalysts

The pristine catalysts were synthesized via a high-temperature vulcanization method using sublimed sulfur as the sulfur source (see the Experimental Section for details). Structural characterization by combined X-ray diffraction (XRD) and transmission electron microscopy (TEM) confirmed the successful formation of the CuS phase (Figures S1–5).^31^ Inductively coupled plasma-atomic emission spectrometry (ICP-AES) analysis revealed a Pd:Cu molar ratio of 0.91% in the Pd_1_/CuS catalyst (Table S1). High-angle annular dark-field scanning transmission electron microscopy (HAADF-STEM) imaging (Figure 1a) resolved the hexagonal lattice structure of CuS in Pd_1_/CuS. X-ray photoelectron spectroscopy (XPS) spectra were employed to investigate the chemical states of main components. As shown in Figure 1b, the Cu 2p spectrum exhibited two distinct peaks at binding energies of ∼932.3 eV (Cu 2p_3/2_) and ∼952.2 eV (2p_1/2_), consistent with the presence of oxidative Cu.^32^ Further Cu LMM Auger analysis of both pristine catalysts showed spectra very similar to those of reported CuS (Figure S6).^33^ The S 2p spectrum displayed two characteristic peaks, corresponding to S^2–^ and S_2_^2–^, in agreement with previous reports on CuS (Figures S7 and S8).^34^ The oxidation state of Pd in Pd_1_/CuS was determined to be predominantly 2+, as evidenced by the Pd 3d peaks at ∼337.2 and ∼342.4 eV (Figure 1c). A subtle yet discernible positive shift (∼0.1 eV) in the Cu 2p binding energy was observed for Pd_1_/CuS compared to that of pristine CuS, suggesting electron loss from Cu upon Pd doping (Figures 1b and S9). This electronic perturbation was further corroborated by Cu L-edge X-ray absorption near-edge structure (XANES) spectroscopy, which revealed an enhanced Cu^2+^ signature relative to that of Cu^+^ (Figure S10). Additionally, Cu K-edge XANES spectra exhibited a positive energy shift in the absorption edge for Pd_1_/CuS, reinforcing the modified electronic environment of the Cu sites (Figure S11).

**Figure 1:**
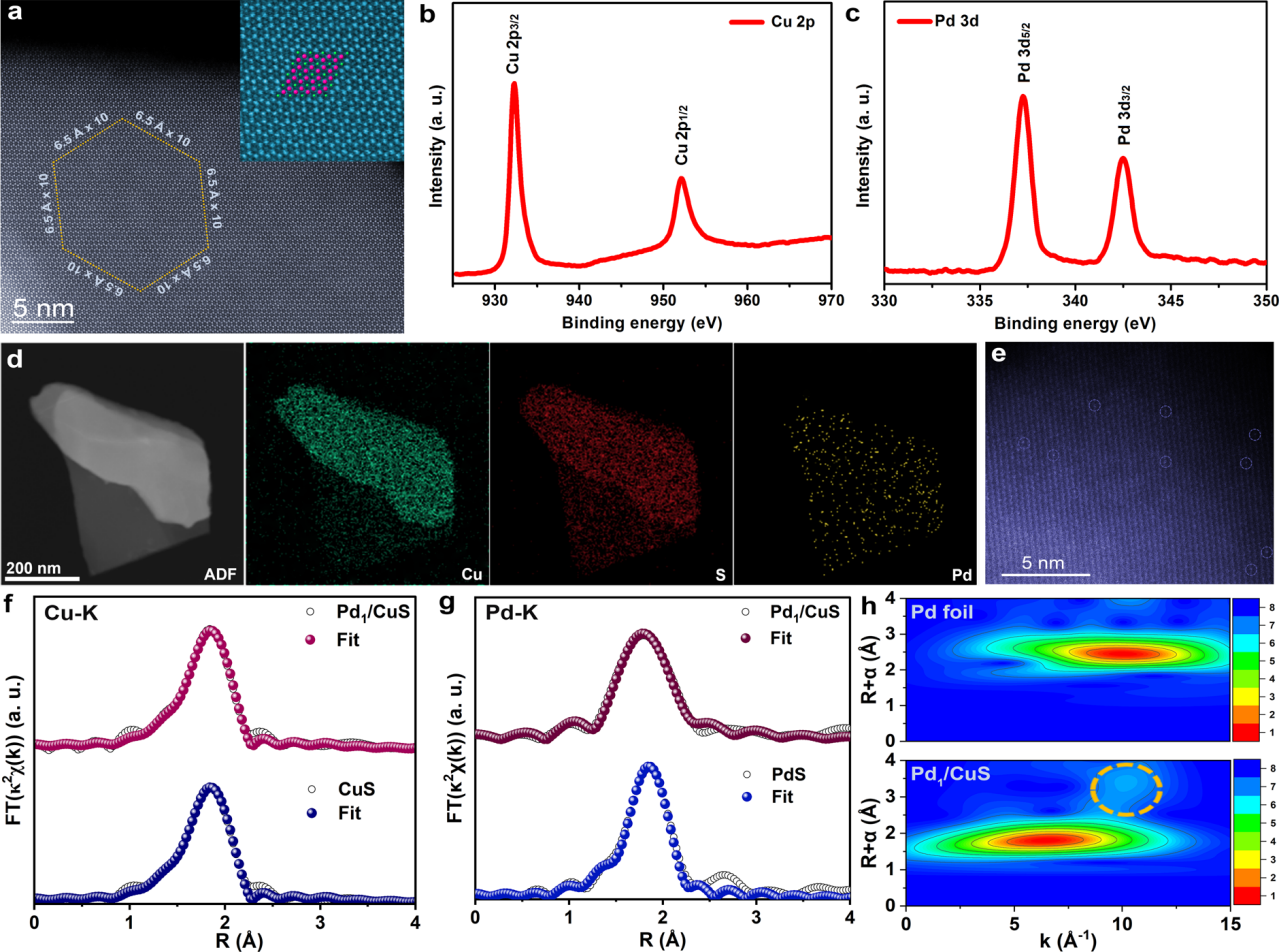
(a) High-angle annular dark-field scanning transmission electron microscopy (HAADF-STEM) imaging of Pd_1_/CuS. High-resolution spectra of (b) Cu 2p and (c) Pd 3d in Pd_1_/CuS. (d) Elemental mappings of Cu, S, and Pd in Pd_1_/CuS. (e) Atomic-resolution HAADF-STEM imaging of Pd_1_/CuS, with circled Pd single-atom sites. (f) Cu K-edge Fourier-transform extended X-ray absorption fine structure (FT-EXAFS) spectra of Pd_1_/CuS and CuS. (g) Pd K-edge FT-EXAFS spectra of Pd_1_/CuS and PdS. (h) Wavelet-transform EXAFS (WT-EXAFS) spectra of the Pd foil and Pd_1_/CuS at the Pd K-edge.

Elemental mappings confirmed the homogeneous distribution of Pd within the CuS matrix (Figure 1d). Atomic-resolution HAADF-STEM imaging revealed the predominant existence of Pd as an isolated site (Figure 1e). Fourier-transform extended X-ray absorption fine structure (FT-EXAFS) analysis and corresponding fitting results indicated that Pd_1_/CuS largely retained the CuS crystal structure, albeit with a slight reduction in Cu–S coordination numbers due to partial substitution of lattice Cu by Pd (Figure 1f and Table S2). Notably, the Pd–S coordination number in Pd_1_/CuS was lower than that in PdS, to enable Pd sites to better match the crystal lattice of CuS (Figure 1g and Table S3). Wavelet-transform EXAFS (WT-EXAFS) analysis further confirmed the preservation of the hexagonal CuS structure after Pd introduction while clearly resolving the Pd-metal scattering signal at ∼10 Å^–1^ in the Pd K-edge spectrum (Figures 1h and S12).

### Electrochemical Performance of Catalysts

The NO_3_RR performances of Pd_1_/CuS and CuS were evaluated in a 1.0 mol L^–1^ potassium hydroxide aqueous solution (1.0 M KOH) containing 0.5 M potassium nitrate (KNO_3_) as the nitrate source. The reference electrode potential was calibrated with a hydrogen reference electrode (Figure S13). Prior to electrochemical testing, all catalysts underwent cyclic voltammetry (CV) activation within suitable potential windows (see the Experimental Section for details). Chronoamperometry measurements were subsequently performed to assess the catalytic performance across a range of applied potentials (Figures S14–16). As illustrated in Figure 2a, activated Pd_1_/CuS maintained exceptional ammonia selectivity (>77.0%) throughout the tested potential range, achieving a near-unity FE (∼100%) at −0.5 V vs RHE. In stark contrast, activated CuS exhibited significantly lower ammonia selectivity (<63.0%) under identical conditions (Figures 2a and S17). Even in electrolytes with a lower nitrate concentration, our catalyst demonstrated considerable performance in the NO_3_RR (Figure S18). The origin of the produced NH_4_^+^ was unequivocally confirmed through ^15^N isotope-labeling experiments, where the characteristic doublet signal of ^15^NH_4_^+^ in ^1^H nuclear magnetic resonance (NMR) spectra exclusively appeared when using ^15^NO_3_^–^ as the reactant, verifying the electrocatalytic origin of the ammonia product (Figure S19).

**Figure 2:**
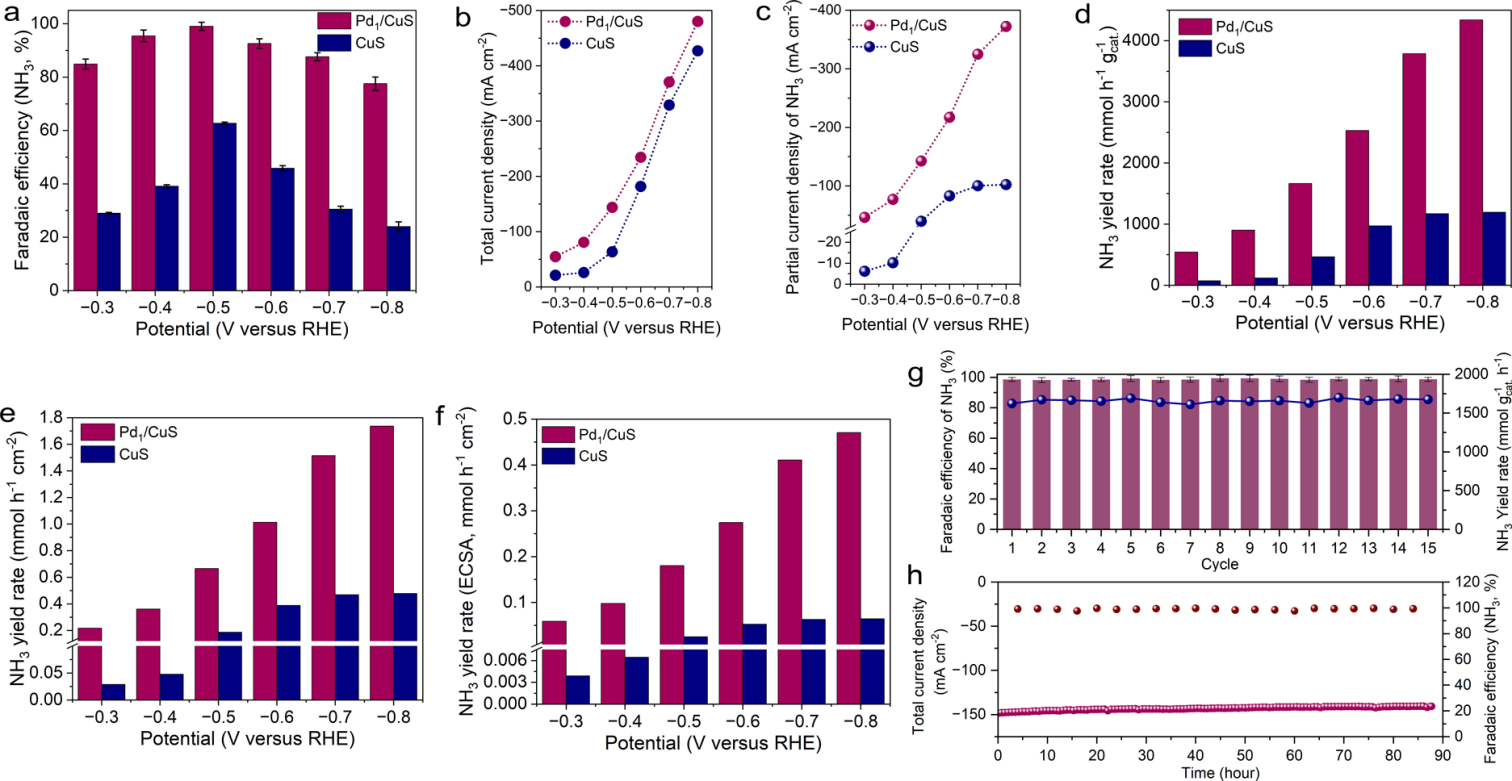
(a) Faradaic efficiency (FE) comparison for ammonia production on Pd_1_/CuS and CuS catalysts across applied potentials. (b) Total current density and (c) NO_3_RR-derived ammonia partial current density profiles for Pd_1_/CuS versus CuS. (d) Mass-normalized ammonia yield rates of both catalysts. Area-normalized yield rates of Pd_1_/CuS and CuS with (e) geometric area and (f) electrochemical surface area (ECSA), respectively. (g) Cycling durability analysis of Pd_1_/CuS at −0.5 V vs RHE. Each cycle lasts 30 min. (h) Long-term stability evaluation of activated Pd_1_/CuS at −0.5 V vs RHE in KOH containing 0.5 M KNO_3_.

Beyond selectivity enhancements, activated Pd_1_/CuS demonstrated superior activity, as evidenced by its substantially higher current densities. The NO_3_RR partial current density for activated Pd_1_/CuS exhibited a continuous increase with more negative potentials, reaching approximately −373 mA cm^–2^ at −0.8 V vs RHE (Figure 2b,c). Conversely, activated CuS showed limited activity (maximum of about −100 mA cm^–2^) with apparent performance saturation at higher overpotentials. These observations underscore the critical role of Pd introduction in enhancing the NO_3_RR performance. Quantitative analysis revealed that activated Pd_1_/CuS achieved a remarkable ammonia yield rate of ∼4350 mmol h^–1^ g_cat_^–1^, outperforming activated CuS (∼1194 mmol h^–1^ g_cat_^–1^) by a factor of 3.6 (Figure 2d). This enhancement is also demonstrated by the geometric area and electrochemical surface area (ECSA) normalized yield rates, with activated Pd_1_/CuS exhibiting 3.6-fold and 7.2-fold improvements, respectively, at −0.5 V vs RHE (Figures 2e,f and S20). The exceptional ECSA-normalized performance particularly highlights the intrinsic performance enhancement from the incorporation of Pd single atoms. This conclusion is further supported by the continuous increase in the FE for ammonia with rising Pd usage and reaches the maximum at a usage of 0.018 mmol (Figure S21). Furthermore, the calculated turnover frequency (TOF) values confirmed the high intrinsic activity of the activated Pd_1_/CuS compared to CuS (Figure S22). Stability assessments confirmed the good durability of activated Pd_1_/CuS, maintaining >98.2% ammonia selectivity and >1600 mmol h^–1^ g_cat_^–1^ yield rate after 15 consecutive cycles at −0.5 V vs RHE (Figures 2g and S23). Long-term electrolysis confirmed the stability of our catalyst, as evidenced by a consistent current density and stable FE for ammonia production (Figure 2h). This good stability, coupled with the enhanced activity and selectivity, established activated Pd_1_/CuS as a superior NO_3_RR catalyst compared to the activated CuS counterpart.

### Experimental Analyses of Structural Evolution

To elucidate the structural evolution of catalysts during NO_3_RR operation, we conducted in situ Raman spectroscopy measurements at −0.7 V vs RHE in a 1.0 M KOH electrolyte containing 0.5 M KNO_3_. As shown in Figure 3a, the characteristic CuS phonon mode at ∼470 cm^–1^ gradually attenuated with reaction time and completely disappeared after about 16 min for Pd_1_/CuS. The absence of new vibrational features during this transformation suggests the electrochemical reduction of CuS to metallic Cu. A similar reduction process was observed for pristine CuS, though with accelerated reduction kinetics evidenced by complete peak disappearance ∼7 min earlier than Pd_1_/CuS (Figure 3b). Complementary in situ electrochemical impedance spectroscopy (EIS) revealed distinct inductive arcs (indicated by the arrows) in the midfrequency region for both catalysts (Figures 3c,d and S24), characteristic of electrode surface reconstruction. The more pronounced inductive signal for CuS compared with Pd_1_/CuS indicates greater structural instability, consistent with the faster reduction kinetics observed by in situ Raman spectroscopy. This enhanced stability of Pd_1_/CuS may be attributed to the strengthening of the Cu–S bonds caused by the doping of Pd. This can be demonstrated by the increased chemical state of Cu in Pd_1_/CuS (Figures S9–11), as well as a more negative crystal orbital Hamilton population (COHP) value of Cu–S in Pd_1_/CuS (Figure S25).

**Figure 3:**
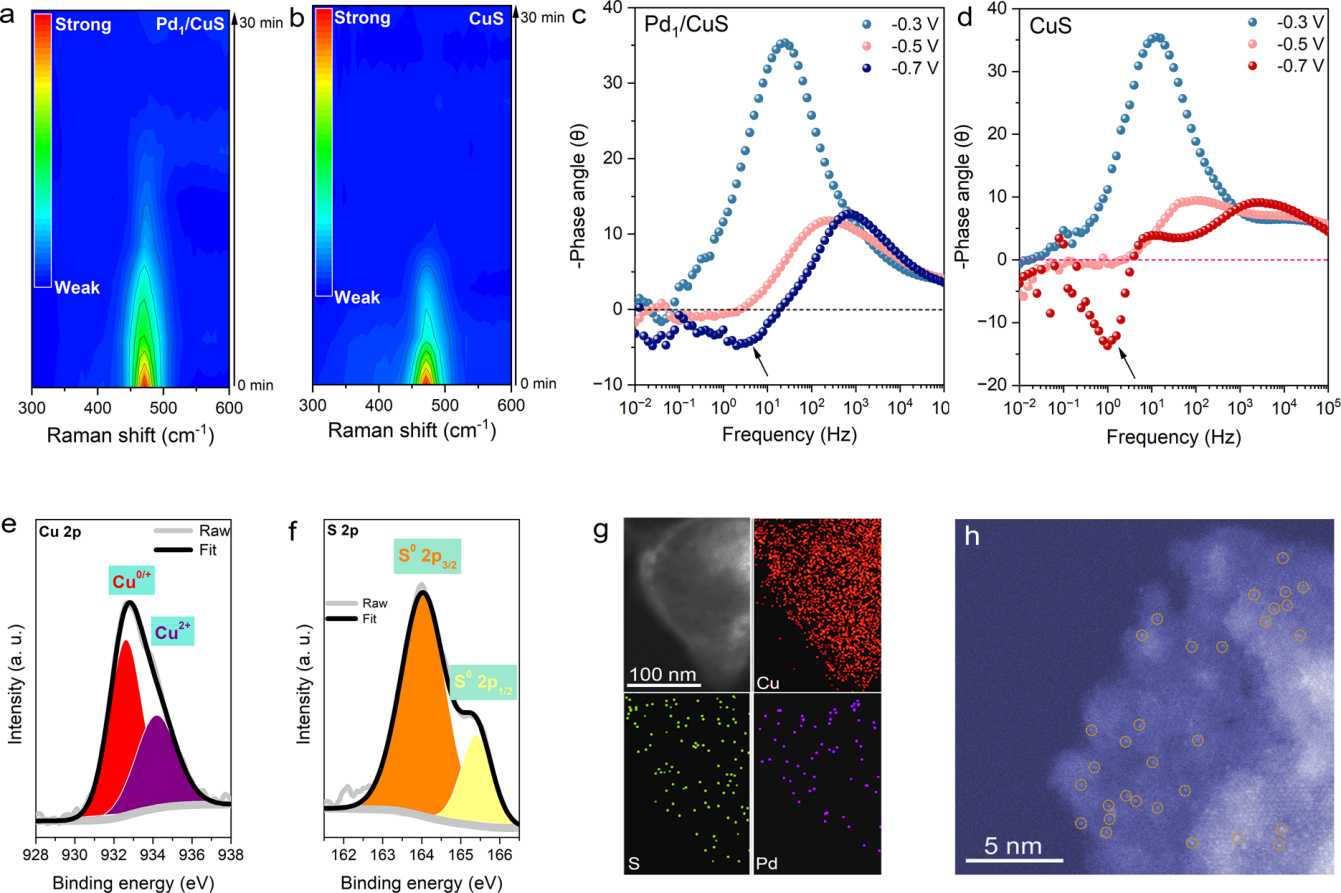
Time-dependent in situ Raman spectra acquired at −0.7 V vs RHE in 1.0 M KOH containing 0.5 M KNO_3_ for (a) Pd_1_/CuS and (b) CuS. Bode plots of (c) Pd_1_/CuS and (d) CuS from in situ EIS measured at various potentials (vs RHE). High-resolution X-ray photoelectron spectroscopy (XPS) of (e) Cu 2p and (f) S 2p of postreaction Pd_1_/CuS. (g) Elemental mappings of Cu, S, and Pd in postreaction Pd_1_/CuS. (h) Atomic-resolution HAADF-STEM imaging of postreaction Pd_1_/CuS.

Postreaction XPS analysis confirmed the coexistence of Cu^0/+^ and Cu^2+^ species, with the oxidative states attributed to the oxidation upon air exposure, as corroborated by the obvious lattice oxygen signal in the O 1s spectrum (Figures 3e and S26–S27). The Cu LMM Auger spectra of the spent catalysts also revealed that Cu species were predominantly in oxidized states (Figure S28).^35^ The S species were predominantly oxidized, indicating complete breakdown of the Cu–S lattice (Figure 3f). Despite the absence of detectable Pd signals, attributed to its low concentration from partial loss and the surface shading by abundant carbon- and oxygen-containing species (Table S1 and Figures S26 and S29), its reduction remains thermodynamically favorable due to its more positive reduction potential compared to CuS.^36^ The residual S exhibited a negligible influence on the performance of the catalyst, as evidenced by the consistent activity and selectivity (Figure S30). Elemental mappings revealed Cu-rich domains with sparse S and Pd distributions (Figure 3g). Crucially, atomic-resolution HAADF-STEM imaging confirmed the preservation of isolated Pd sites after electrolysis, demonstrating that the isolated Pd sites modified Cu as the active configuration for NO_3_RR electrocatalysis (Figure 3h).

### Mechanistic Study Based on In Situ Multiple Spectroscopies

Through comprehensive electrochemical and spectroscopic analyses, we elucidate the mechanistic origin of the enhanced NO_3_RR performance on a Pd-modified Cu catalyst. CV characterization reveals distinct redox behaviors of Pd_1_/CuS and CuS (Figure 4a,b). Both catalysts undergo structural transformation during the initial several cycles, evidenced by (feature A) the reduction of CuS to metallic Cu, occurring more rapidly for CuS (consistent with in situ Raman and Bode plot results); (feature B) concurrent oxidation of lattice S to S^0^; and (feature C) gradually increasing reduction current density. These observations confirm that the reconstructed surfaces exhibit superior catalytic activity compared to the pristine materials. Notably, the CV profiles show fundamental differences in reaction characteristics between the two catalysts. For CuS, the persistent reduction feature (feature D) indicates adsorption-limited surface reactions where excess reaction intermediates, such as *NO_3_, dominate the surface sites (Figure 4c). In contrast, Pd_1_/CuS exhibits a negative potential shift in the reduction peak (feature D), along with peak broadening and intensification. These indicate the enhanced conversion of adsorbed intermediates on the modified heterogeneous active sites due to Pd incorporation. Additionally, there is a positive shift in the reduction onset potential (feature E), suggesting a facilitated NO_3_RR.

**Figure 4:**
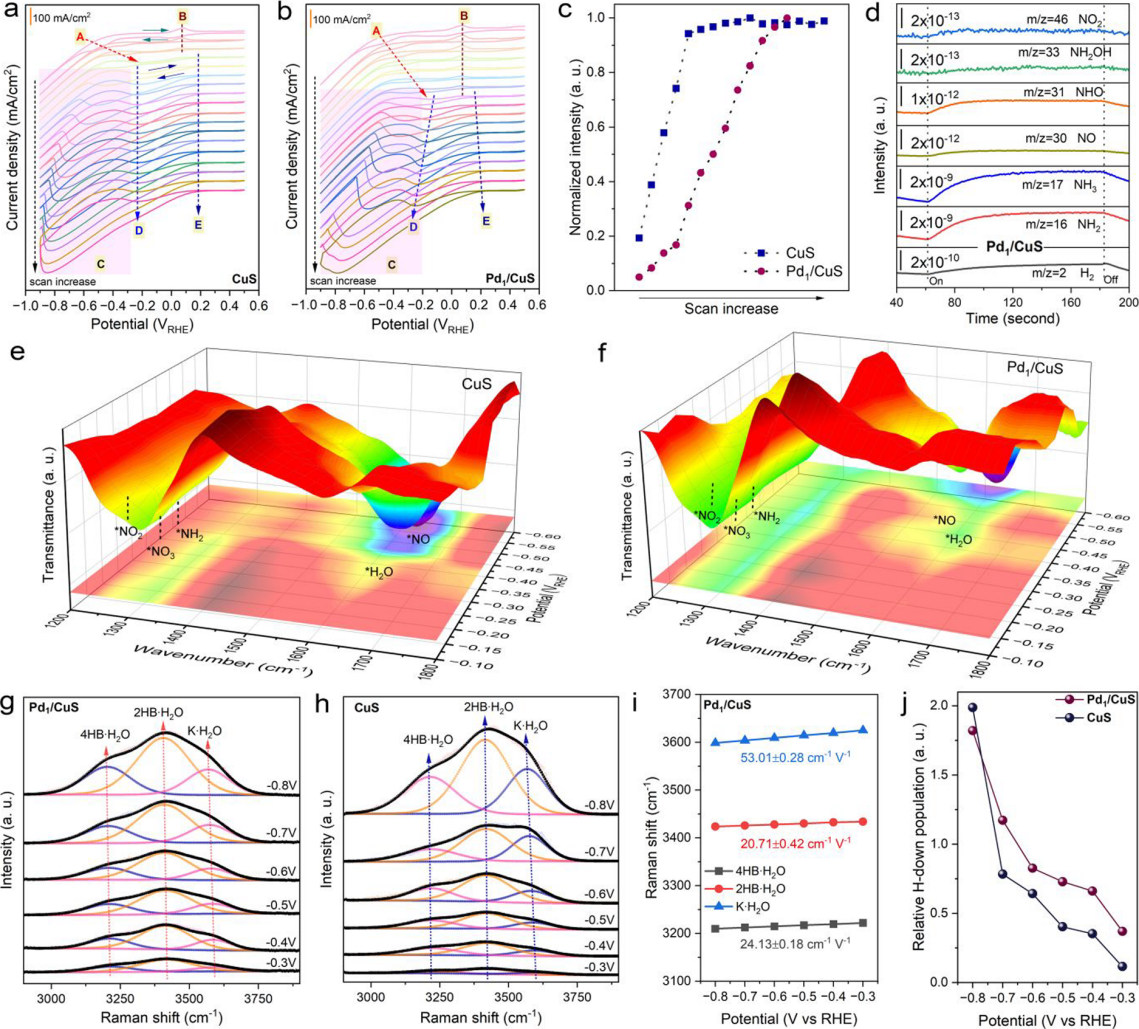
Cyclic voltammetry (CV) curves of (a) CuS and (b) Pd_1_/CuS collected at potential windows of +0.5 to −0.9 V vs RHE in 1.0 M KOH containing 0.5 M KNO_3_. (c) Normalized intensity of peak D in (a, b) with the highest peak as the denominator for CuS and Pd_1_/CuS, respectively. (d) Online differential electrochemical mass spectrometry (DEMS) of activated Pd_1_/CuS. In situ synchrotron radiation infrared (SR-IR) spectra of activated (e) Pd_1_/CuS and (f) CuS. In situ Raman spectra of activated (g) Pd_1_/CuS and (h) CuS with fitting of the O–H stretching mode band of interfacial water. (i) Potential-dependent Stark effect shift analysis from (g). (j) Comparison of the relative H-down population for activated Pd_1_/CuS and CuS.

Online differential electrochemical mass spectrometry (DEMS) analysis of activated catalysts identified key reaction intermediates (*NH_2_, *NH_3_, *NO, *NHO, *NH_2_OH, and *NO_2_), validating the proposed reaction pathway: *NO_3_^–^ → *NO_2_^–^ → *NO → *NHO → *NH_2_OH → *NH_3_ (Figures 4d and S31). This mechanistic understanding offers valuable insight into the catalytic cycle. Furthermore, in situ synchrotron radiation infrared (SR-IR) spectroscopy measurements identified key intermediates such as *NH_2_, *NO, *NO_2_, and *NO_3_, providing additional support for the proposed reaction pathway (Figure 4e,f). Notably, both activated catalysts exhibited pronounced *NO_2_ and *NO_3_ peaks at high potentials, indicating a strong adsorption capacity for these intermediates. However, for activated CuS, the *NO_2_ peaks relative to *NO_3_ significantly weakened at lower potentials, suggesting low efficiency in the *NO_3_ to *NO_2_ conversion. This excessive adsorption of *NO_3_ might limit surface reactions on activated CuS, as indicated by the CV results. In contrast, activated Pd_1_/CuS displayed continuous and distinct peaks for *NH_2_, *NO_2_, and *NO_3_ across the measured potential range, suggesting efficient and ongoing conversion among intermediates. The relative intensities of peaks corresponding to the bending mode of adsorbed water (*H_2_O) were stronger for activated Pd_1_/CuS compared to that for activated CuS, indicating more pronounced hydrophilicity. This hydrophilicity is advantageous for the adsorption of hydrophilic species such as H_2_O and H^+^. Another point of interest is the emergence of the *NO peak. Under very negative potentials, activated CuS showed dominant *NO peaks at higher wavenumbers, whereas activated Pd_1_/CuS exhibited pronounced *NO peaks at relatively lower wavenumbers. Additionally, the relative intensity of *NO to *H_2_O was lower for activated Pd_1_/CuS. These observations highlight that the incorporation of Pd markedly influences the reaction processes, enhancing the adsorption of intermediates in the NO_3_RR and improving the proton supply to facilitate the deep reduction of nitrate.

Active hydrogen (*H) is recognized as a critical factor affecting the selectivity of the nitrate reduction reaction (NO_3_RR).^37^ To gain deeper insight into the kinetic behavior of surface-active hydrogen species during the NO_3_RR, we conducted H/D kinetic isotope effect (KIE) studies to evaluate the contribution of proton transfer in the rate-determining step of the electrocatalytic reaction. In our experiments, replacing H_2_O with D_2_O led to a significant decrease in the NO_3_RR rates for both catalysts, underscoring the essential role of *H in the reaction process (Figure S32). The lower KIE value observed for activated Pd_1_/CuS compared to that for activated CuS indicates accelerated hydrogen transfer kinetics in activated Pd_1_/CuS, confirming that Pd single-atom doping facilitates water dissociation and enhances the supply of *H, which aligns with the DFT calculations. Considering that interfacial H_2_O acts as a major source of *H under alkaline conditions, we further employed in situ Raman spectroscopy to probe the molecular-level behavior of water at the electrode–electrolyte interface. This approach aimed to deepen our understanding of how the introduction of Pd enhances the NO_3_RR performance of the catalyst. Raman spectra acquired at various potentials for activated Pd_1_/CuS and CuS are shown in Figure 4g,h. A broad Raman band, attributed to the O–H stretching mode of H_2_O, was detected in the range of 3000 to 3800 cm^–1^. The peak intensity for both activated Pd_1_/CuS and CuS increased with a decrease in potential, indicating a dynamic, potential-dependent interfacial structure. Further deconvolution of the interfacial H_2_O band revealed three distinct peaks, corresponding to three types of H_2_O molecules: alkali metal ion hydrated H_2_O (K·H_2_O) at approximately ∼3600 cm^–1^, 2-coordinated hydrogen-bonded H_2_O (2HB·H_2_O) at ∼3423 cm^–1^, and 4-coordinated hydrogen-bonded H2O (4HB·H_2_O) at ∼3218 cm^–1^. Analysis of the vibrational Stark effect, as reflected in potential-dependent Raman shifts, revealed that the K·H_2_O peak was more sensitive to applied potentials than the 2HB·H_2_O and 4HB·H_2_O peaks, which exhibited similar slopes (Figures 4i and S33). Notably, the slope of the K·H_2_O peak for activated Pd_1_/CuS was significantly smaller than that for activated CuS. Furthermore, normalized intensity analyses indicated that the proportion of 2HB·H_2_O was dominant in both activated catalysts, but it was more significant in activated Pd_1_/CuS (Figure S34). This suggests that interfacial K·H_2_O molecules over activated Pd_1_/CuS were more prone to partial desolvation. Partial desolvation of K·H_2_O allows it to approach the surface more closely and partially shield the local electric field from external potential, thereby stabilizing intermediates with dipole moments in NO_3_RR, as revealed by in situ SR-IR results.^38^ Additionally, partially desolvated K^+^ may stabilize the transition state of the Volmer step (Volmer: H_2_O → *H + OH^–^), serving as a proton source for the hydrogenation processes occurring in NO_3_RR.^39,40^ Normalized proportion analyses indicated that at potentials between −0.4 and −0.6 V vs RHE, activated Pd_1_/CuS exhibited a comparable proportion of K·H_2_O to activated CuS, while activated CuS had a significantly higher proportion of K·H_2_O at other measured potentials (Figure S35). Additionally, the relative H-down population of activated Pd_1_/CuS was higher than that of activated CuS (Figure 4j).^41^ However, with further potential decrease to −0.8 V vs RHE, the H-down population of activated Pd_1_/CuS became slightly lower than that of activated CuS. Given the relatively higher ammonia selectivity at −0.4 to −0.6 V vs RHE for activated CuS, as well as the consistently high ammonia selectivity at measured potentials for activated Pd_1_/CuS, it is suggested that the enhanced selectivity conferred by Pd introduction may partially originate from a positive regulation of the interfacial microenvironment. Generally speaking, the partially desolvated K·H_2_O being closer to the electrode stabilizes the intermediates in the NO_3_RR and promotes water dissociation to accelerate proton supply. Additionally, an enriched H-down interfacial water population establishes a strong hydrogen-bond network for proton transfer. As a result, accelerated transfer of *H ensures the efficient conversion of stably adsorbed intermediates in the NO_3_RR, favoring the ammonia-selective pathway. Although at −0.8 V vs RHE, the H-down population is higher for activated CuS than for activated Pd_1_/CuS, the electrochemical ammonia selectivity of activated CuS remains significantly lower. This result further implies that microenvironment regulation over the electrode surface is not the sole determinant of electrochemical performance. Key elementary steps in surface reactions, including the NO_3_RR and side reactions such as the HER, are also crucial for performance, as revealed in the following theoretical calculation section.

Considering the findings presented above, the introduction of Pd appears to enhance the adsorption of reactants and intermediates on the catalyst by promoting the partial desolvation of hydrated alkali metal ions. This, in turn, improves the dissociation of water and hydrogenation of intermediates in NO_3_RR. Additionally, the interfacial proton transfer is facilitated by strengthening the hydrogen-bond network. These combined effects contribute to the high ammonia selectivity observed for activated Pd_1_/CuS.

### Theoretical Analysis

Our experimental findings demonstrated that pristine sulfide precatalysts undergo in situ transformation into pure Cu and isolated Pd-doped Cu catalysts under reaction conditions. To investigate the role of Pd in enhancing the NO_3_RR performance on Cu, we conducted density functional theory (DFT) to analyze both NO3RR and HER processes. First, we calculated the reaction free-energy profiles for nitrate conversion (Figures 5a and S36). Both Cu and Pd-doped Cu exhibited similar reaction energy barriers at the potential determining steps (PDS): 0.317 eV for the *NH_2_O → *NH_2_OH step and 0.329 eV for the *NO → *NHO step, indicating that protonation processes were not significantly affected by Pd doping. However, nitrate adsorption on Pd-doped Cu (0.461 eV) was weaker compared to Cu (0.330 eV). These analyses suggest that Pd doping does not confer a thermodynamic advantage for surface reactions involving the NO_3_RR, implying that the conclusions from experimental analysis likely stem from interfacial regulation. This emphasizes the importance of interfacial properties in our electrocatalytic process.

**Figure 5:**
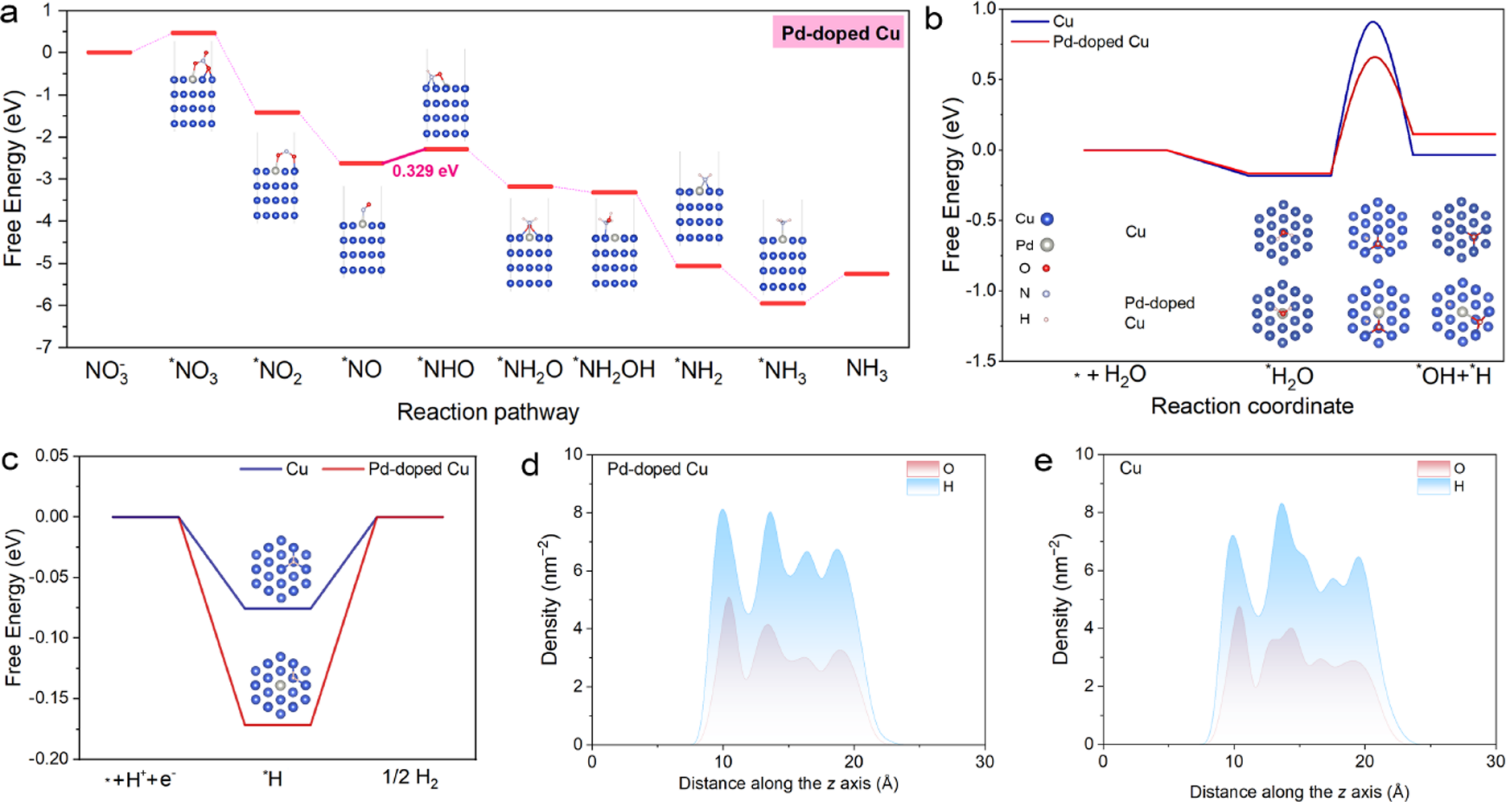
(a) Reaction Gibbs free energy of NO_3_RR and the optimized structures of the reaction intermediates on Pd-doped Cu. (b) Free-energy profiles and the optimized structures of H_2_O into *H on Cu and Pd-doped Cu. (c) Free-energy diagrams along with the optimized intermediate for HER. The density distribution of the O and H atoms along the *z* axis on Pd-doped Cu (d) and Cu (e) catalysts.

Interestingly, Pd doping showed a preference for capturing H_2_O molecules through an exothermic process, with a free-energy difference (−0.165 eV) close to that of Cu (−0.183 eV) (Figure 5b). Further analysis revealed that the dissociation of *H_2_O into active *H intermediates occurred with a lower kinetic barrier on Pd-doped Cu (0.82 eV) compared with Cu (1.09 eV). HER calculations indicated that Pd-doped Cu exhibited a higher energy barrier for H_2_ formation (0.17 eV vs 0.08 eV) from the *H intermediate, suggesting that isolated Pd atoms not only kinetically promoted the generation of *H intermediates but also thermodynamically suppressed competing HER processes (Figure 5c). Additionally, AIMD simulations at 300 K were performed to examine the impact of Pd doping on the interfacial microenvironments. According to Bader charge analysis (Figure S37), Pd acquired approximately 0.37 e^–^ from its surrounding environment, resulting in a negatively charged state (Pd^δ–^). We further investigated the influence of Pd^δ–^ species on the distribution of H_2_O molecules along the surface normal of the catalysts. AIMD simulations qualitatively revealed that the average number of hydrogen bonds per water molecule on the Pd-doped surface was lower than that on the Cu surface (indicated by the blue box), suggesting a higher proportion of 2HB·H_2_O, which facilitates the proton-transfer process (Figure S38). The first peak corresponding to H atoms appeared earlier than that for the O atoms on both Cu and Pd-doped Cu catalysts, indicating a predominance of H-down configurations (Figures 5d,e and S39–S42). Furthermore, the population density of H-down hydrogens on Pd-doped Cu was significantly higher compared to Cu, highlighting the pronounced effect of Pd on interfacial water dipole orientations, consistent with in situ Raman results. Consequently, the enriched *H species transfer through an enhanced hydrogen-bond network facilitates the hydrogenation process in the NO_3_RR for ammonia production.

## Conclusions

In summary, this work systematically investigated the catalytic mechanism of a Pd-doped CuS precatalyst for NO_3_RR. By integrating in situ spectroscopic techniques, microscopic analysis, and theoretical simulations, we demonstrated that the precatalyst undergoes dynamic electrochemical transformation into a metallic phase under operating conditions while preserving the isolated Pd sites. This reconstructed catalyst exhibited high performance, achieving a near-unity FE and high ammonia yield rates, significantly outperforming its undoped counterpart. Mechanistic studies revealed that Pd incorporation not only enhances water dissociation to promote proton supply but also suppresses the HER by increasing the energy barrier for *H recombination thermodynamically. Furthermore, Pd incorporation facilitates the partial desolvation of hydrated alkali ions, promoting water dissociation and increasing the H-down water population to strengthen the hydrogen-bond network for proton transfer, thereby favoring the ammonia-selective pathway. This study provides fundamental insights into the correlation between dynamic structural evolution and catalytic performance, highlighting the critical influence of structural regulation on the reaction microenvironments. These findings not only deepen the mechanistic understanding of NO_3_RR on Cu-based catalysts but also offer strategic guidelines for the rational design of high-performance electrocatalysts through structural engineering.

## Supplementary Material



## Notes

Experimental section and characterization details; additional characterization spectra and electrochemical data; and theoretical details (PDF)
