# Supplementary material for: Dynamic Reconstruction and Microenvironment Modulation of a Pd-Doped CuS Electrocatalyst for Nearly Unity-Efficiency Ammonia Electrosynthesis from Nitrate
Source: J Am Chem Soc. 2025 Nov 6;147(46):43067–76. doi: 10.1021/jacs.5c16232 (PMC12817242; doi:10.1021/jacs.5c16232)
Supplement: Supplementary file 1 [file ja5c16232_si_001.pdf]

Supplementary Information

for

Dynamic Reconstruction and Microenvironment Modulation of a Pd-Doped CuS Electrocatalyst for Nearly Unity-Efficiency Ammonia Electrosynthesis from Nitrate

Qun He,[†,§] Zhangsheng Shi,[†,§] Dongxue Yu,[†] Yunpeng Zuo,[†] Wei Jiang,[‡] Hengjie Liu,[‡] Chuanqiang Wu,[#] Li Song,[‡] and Xin Wang,[†,*]

[†] Dr. Q. He, Z. Shi, D. Yu, Dr. Y. Zuo, Prof. X. Wang

Department of Chemistry

City University of Hong Kong

Kowloon 999077, China

Email: wang.xin@cityu.edu.hk

[‡] Dr. W. Jiang, Prof. L. Song

National Synchrotron Radiation Laboratory

University of Science and Technology of China

Hefei 230029, China

[#] Prof. C. Wu

Information Materials and Intelligent Sensing Laboratory of Anhui Province, Key Laboratory of Structure and Functional Regulation of Hybrid Materials of Ministry of Education, Institutes of Physical Science and Information Technology, Anhui University, Hefei 230601, China

[§] These authors contributed equally to this work.

Experimental Section

Synthesis of Pd_1/CuS and CuS. A homogeneous solution was prepared by dissolving 1.5 mmol of $CuCl_2 \cdot 2H_2O$ and 0.018 mmol of K_2PdCl_4 in 4.5 mL of ethanol under continuous stirring for 30 minutes. Subsequently, 1.3 mL of (±)-propylene oxide was added to the mixture, and the reaction was allowed to proceed for 24 hours. The resulting product was collected via centrifugation, followed by vacuum drying at 60 °C. The dried powder was then placed at the bottom of a quartz boat and completely covered with sublimed sulfur. Prior to annealing, the quartz tube was purged with argon (200 sccm) for one hour to ensure an oxygen-free environment. The quartz boat was heated to 400 °C at a ramp rate of 2 °C min^{-1} and maintained at this temperature for 3 hours. It should be noted that the exhaust gas needs to be absorbed by sodium hydroxide solution. After annealing, the obtained powder was thoroughly ground and sequentially washed with carbon disulfide, deionized water, and ethanol (three times each). Finally, the Pd_1/CuS was obtained after vacuum drying at 60 °C. The synthesis of CuS and Pd-doped CuS with various contents followed a similar process to that of Pd_1/CuS, with the exception of the addition of K_2PdCl_4 or the usage amount change of K_2PdCl_4 to 0.006, 0.012, and 0.03 mmol.

Characterization Methods. The crystallographic structures of the samples were characterized by X-ray diffraction (XRD) using a Rigaku SmartLab X-ray diffractometer with Cu K_α radiation. Transmission electron microscopy (TEM) and high-resolution TEM (HRTEM) were performed on a JEM-2100F field-emission electron microscope operated at 200 kV to examine the morphology and microstructure. Atomic-scale imaging was conducted via high-angle annular dark-field scanning TEM (HAADF-STEM) using a JEOL JEM-ARF200F (200 kV) equipped with a spherical aberration corrector, coupled with energy-dispersive X-ray spectroscopy (EDS) for elemental mapping. The chemical states of the elements were analyzed by X-ray photoelectron spectroscopy (XPS) on a Thermo ESCALAB 250 spectrometer with a monochromatic Al K_α X-ray source (1486.6 eV). All binding energies were referenced to the C 1s peak (284.8 eV) for charge correction. ^{1}H nuclear magnetic resonance (NMR) spectra were recorded on a Bruker AVANCE III 400 MHz spectrometer. Inductively coupled plasma atomic emission spectroscopy (ICP-AES, PerkinElmer Optima 7300 DV) was employed to determine the metal loading concentrations. X-ray absorption spectroscopy (XAS), including Cu L-edge XANES, Cu K-edge XAFS, and Pd K-edge XAFS, was conducted at Hefei Light Source (HLS, beamline XMCD), Beijing Synchrotron Radiation Facility (BSRF, beamline 1W1B), and Shanghai Synchrotron Radiation Facility (SSRF, beamline 14W1), respectively.

Electrochemical Measurements. The electrochemical measurements were performed using an MULTI AUTOLAB M204 electrochemical workstation with a standard three-electrode system (H-cell), consisting of a catalyst loaded carbon paper working electrode (0.5 cm^2), carbon rod counter electrode, and Hg/HgO reference electrode (calibrated against RHE) in an Ar-saturated 1.0 M KOH + 0.5 M KNO_3 electrolyte. The reference electrode potential was calibrated with hydrogen reference electrode (PHY-RHE). Catalyst inks were prepared by ultrasonically dispersing 2 mg sample in a mixture of 0.50 mL deionized water, 0.40 mL isopropyl alcohol, and 0.10 mL 0.5 wt% Nafion solution for 1 hour, with 100 μL of the homogeneous ink drop-cast onto carbon paper and dried at room temperature. Prior to testing, catalysts were activated through 100 cyclic voltammetry (CV) cycles (0.5 to -0.7 V vs. RHE, 100 mV s^{-1}) to achieve stable performance. Electrochemical characterization included CV analysis at 5 mV s^{-1} to examine evolution processes, chronoamperometry tests (-0.3 to -0.8 V vs. RHE) for performance evaluation, and product selectivity determination by UV-Vis spectrophotometry and gas chromatography. Stability was assessed via chronoamperometry at -0.5 V vs. RHE, while electrochemical surface areas were determined from non-Faradaic CV scans for calculating intrinsic activities. Mass activity was determined based on catalyst loading. Additional electrochemical impedance spectroscopy (EIS) measurements were conducted at various potentials to analyze electrochemical process characteristics.

Analysis of Liquid Products. For ammonia quantification, 2 mL of the diluted electrolyte product was mixed with 2 mL of a 1.0 M NaOH solution containing 5 wt% salicylic acid and 5 wt% sodium citrate. Subsequently, 1 mL of 0.05 M NaClO and 0.2 mL of 1 wt% sodium nitroferricyanide solution were added. The mixture was vortexed to ensure homogeneity and then aged for 2 hours. The absorption spectrum of the resulting solution was measured using a UV-Vis spectrophotometer, with the absorbance peak at approximately 652 nm used for quantitative analysis. For nitrite quantification, 2 mL of the diluted electrolyte product was mixed with 2 mL of Griess reagent. The Griess reagent was prepared by dissolving 1.0 g of sulfanilamide, 0.1 g N-(1-naphthyl) ethylenediamine dihydrochloride, and 3.0 mL of H_3PO_4 in 100 mL deionized water. After mixing, the solution was stored in the dark for 30 minutes and the absorbance at approximately 540 nm was recorded for calculation.

The Faradaic efficiency (FE) for the production of ammonia or nitrite was calculated using the following equation:

$$FE = N \times c \times V \times F / (M \times Q \times 10^6)$$

where:

N: the number of electrons transferred per product molecule or ion (8 for NH_3, 2 for NO_2^-),

c: the measured product concentration (ug mL^{-1}),

V: the volume of the electrolyte (mL),

F: Faraday constant (96485.3 C mol^{-1}),

M: the molar mass (ammonia is 17 g/mol, nitrite is 46 g/mol),

Q: the total charge passed during electrolysis (C).

The mass-based and geometric area-based ammonia yield rates were calculated as:

$$v_{mass} = c(NH_3) \times V \times F \times 10^{-3} / (M \times t \times m)$$

$$v_{area} = c(NH_3) \times V \times F \times 10^{-3} / (M \times t \times S)$$

where:

t: the total electrolysis time,

m: the mass of the catalyst loaded,

S: the effective geometric area of the electrode.

Turnover Frequency Calculations. The turnover frequency (TOF) values of ammonia conversion for catalysts are calculated based on the following procedure.

For Cu(111) facet, its atomic density is approximately 17.5 atoms/nm^2. Due to the low concentration of Pd, it did not be considered when assessing the atomic density based on ECSA analysis. ECSA results suggested that activated CuS and Pd_1/CuS had capacitances of 0.297 and 0.1475 mF, respectively. Based on a reference of 40 μF/cm^2, the estimated area of activated CuS and Pd_1/CuS are 7.425 and 3.6875 cm^2. Therefore, the atomic numbers for activated CuS and Pd_1/CuS should be approximately 1.3×10^{16} and 6.5×10^{15} atoms. Then, the TOF values can be calculated using the following equation:

$$TOF\ (h^{-1}) = A_{area} \times S \times N_A / (n \times 10^3)$$

where:

A_{area}: geometric area-based ammonia yield rates (Figure 2e)

S: the effective geometric area of the electrode (0.5 cm^2).

N_A: Avogadro's constant (6.02×10^{23}/mol)

n: surface atomic number

Isotopic $^{15}NO_3^-$ Labelling Tests. The electrolysis was conducted with 0.5 M $K^{15}NO_3$ at -0.5 V_{RHE} for 15 minutes. Firstly, 0.4 mL of the product-containing electrolyte was mixed with 0.3 mL deionized water and 0.3 mL 2 M HCl. Then, 0.5 mL of the mixed solution, 0.04 mL Dimethyl sulfoxide (DMSO) and 0.5 mL deuterium water (D_2O) were homogeneously mixed and transferred into the NMR tube for ^1H NMR test.

In-Situ DEMS, Raman and SR-IR Tests. The in-situ DEMS measurements were conducted using a LingLu QAS100 instrument (Linglu, China) in a three-electrode electrochemical cell, comprising a catalyst-coated working electrode, a Pt wire counter electrode, and an Ag/AgCl reference electrode. The electrolyte consisted of 0.5 M KNO_3 in 1.0 M KOH. Prior to testing, the pristine catalysts were activated via CV scans. Subsequently, chronoamperometry tests were performed at designated potentials after the baseline stabilized. Differential mass signals corresponding to H_2 (2), NH_2 (16), NH_3 (17), NO (30), NHO (31), NH_2OH (33), and NO_2 (46) were detected on the electrode surface.

In-situ Raman spectra were acquired using a Horiba XploRA Plus confocal Raman microscope equipped with a 1200 grooves/mm diffraction grating. A 638 nm laser with an output power of 30 mW served as the excitation source. The acquisition time is 10 seconds and each spectrum accumulate 3 times. The filter is set at 50%. Calibration was performed using a silicon wafer standard at 520 cm^{-1}, and spectral data were collected using a CCD detector (1024 × 256 pixels). Measurements were carried out within a potential range of -0.3 V to -0.8 V vs. RHE, with a step size of 0.1 V. All electrochemical tests were performed in an Ar-saturated 1.0 M KOH electrolyte containing 0.5 M KNO_3.

In-situ SR-IR analysis was performed at the BL01B infrared beamline of the Hefei Light Source (HLS), employing a Bruker Vertex 80v FT-IR spectrometer with a liquid nitrogen-cooled mercury-cadmium-telluride (MCT) detector. Each infrared absorption spectrum was recorded by averaging 128 scans at a resolution of 4 cm^{-1}. Prior to data acquisition, a background spectrum was collected at open-circuit potential for baseline correction. The potential window spanned from -0.10 V to -0.60 V vs. RHE, with increments of 0.05 V. All electrochemical tests were conducted in an Ar-saturated 1.0 M KOH electrolyte with 0.5 M KNO_3.

DFT Calculations and AIMD Simulations. Density-functional theory (DFT) Calculations and Ab Initio Molecular Dynamics (AIMD) Simulations: All the calculations based on the density functional theory (DFT) methods

were performed by Ab initio Simulation Package (VASP)[1,2] with the projector augmented wave (PAW) method.[3] The generalized gradient approximation (GGA) of Perdew-Burke-Ernzerhof (PBE) function was utilized to describe the exchange-correlation interactions.[4] A kinetic energy cutoff of 500 eV was used for all calculations. The convergence threshold was set as 10^{-5} eV for energy and 0.01 eV/Å for force. The smearing width for gaussian smearing was set as 0.10 eV. All surfaces based on a 4-layer 4 × 4 supercell of Cu (111) termination were modeled. The bottom two layers were fixed as bulk regions, and everything else is allowed to relax. A vacuum layer of at least 20 Å was included along the z direction to avoid the interaction between adjacent images. A k-point mesh of 3 × 3 × 1 was used for both structural relaxation and self-consistent calculation. The zero damping DFT-D3 method of Grimme was applied for dispersion correction.[5] VESTA was adopted to show the structures,[6] and the VASP calculation data was dealt with the post-processing VASPKIT package.[7] The transition state searches were conducted by combining the climbing image-nudged elastic band (CI-NEB) and dimer methods.[8] Vibrational frequencies were analyzed to confirm the transition state with only one imaginary frequency. The Gibbs free energy changes were evaluated according to $\Delta G=\Delta E+\Delta ZPE-T\Delta S$, where ΔE is the DFT total energy difference, ΔZPE is the zero-point energy correction, and $T\Delta S$ accounts for entropic contributions. Ab initio molecular dynamics (AIMD) simulations were conducted using the CP2K/Quickstep package.[9] A supercell consisting of 128 Cu atoms, 4 K^+ cations, 4 NO_3^- anions, and 108 O water molecules were constructed to simulate the interfacial solvent/catalyst systems. The canonical ensemble condition (NVT) was imposed by a Nose-Hoover thermostat at a constant temperature of 300 K. The total simulation time was 20 ps to ensure the equilibration of interfacial systems and the timestep was set to 1fs. The post-processing for the spatial distribution of solvent molecules along the z direction was completed by VMD software.[10]

Supplementary Figures and Tables

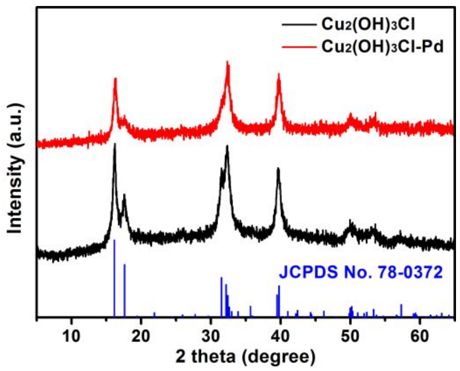

Figure S1. XRD patterns of $Cu_2(OH)_3Cl$ and $Cu_2(OH)_3Cl$-Pd precursors.

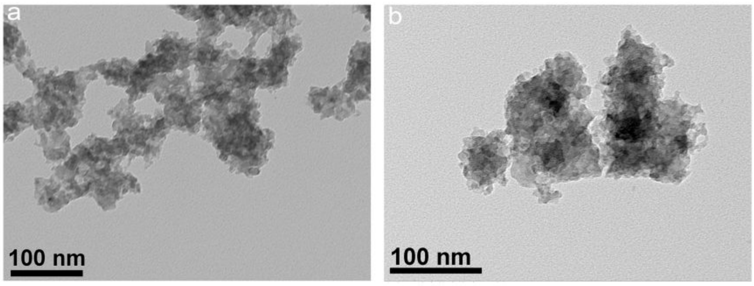

Figure S2. TEM images of (a) $Cu_2(OH)_3Cl$ and (b) $Cu_2(OH)_3Cl$-Pd precursors.

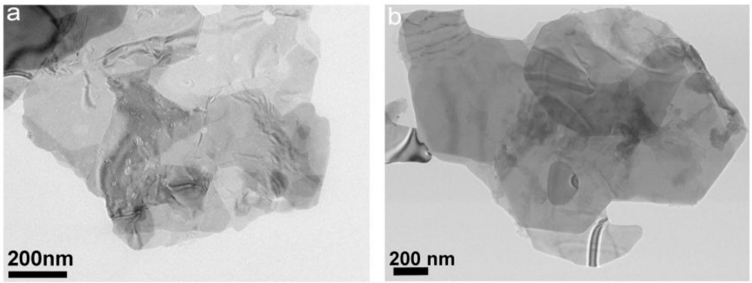

Figure S3. TEM images of (a) CuS and (b) Pd_1/CuS.

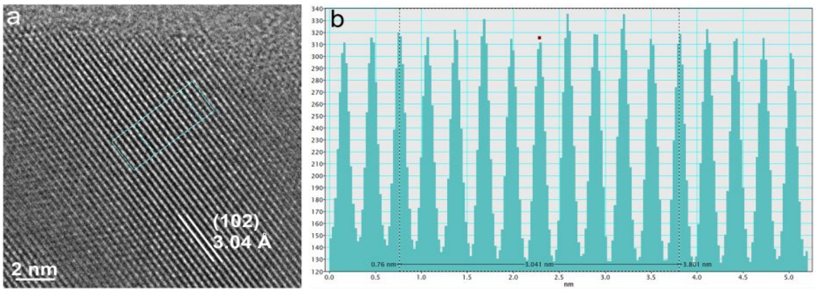

Figure S4. (a) HRTEM and (b) height profile of CuS.

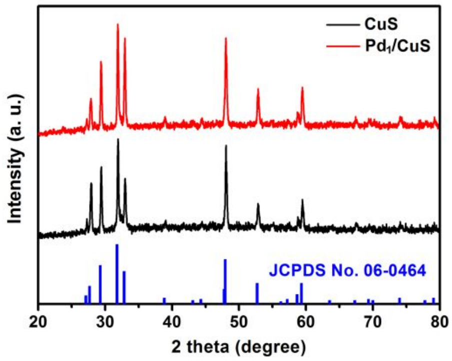

Figure S5. XRD patterns of CuS and Pd_1/CuS.

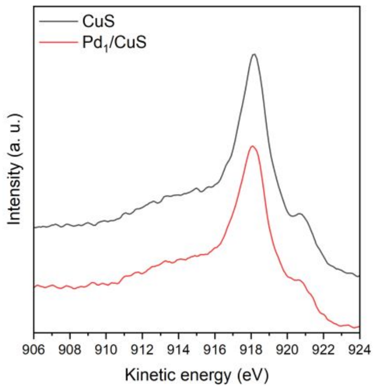

Figure S6. Cu LMM Auger spectra of pristine CuS and Pd_1/CuS.

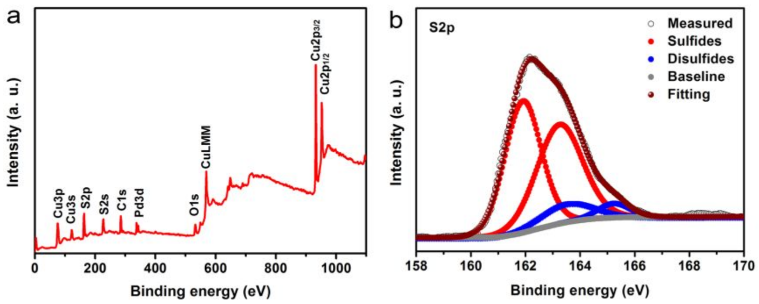

Figure S7. (a) XPS survey and (b) high-resolution S 2p spectrum of Pd_1/CuS.

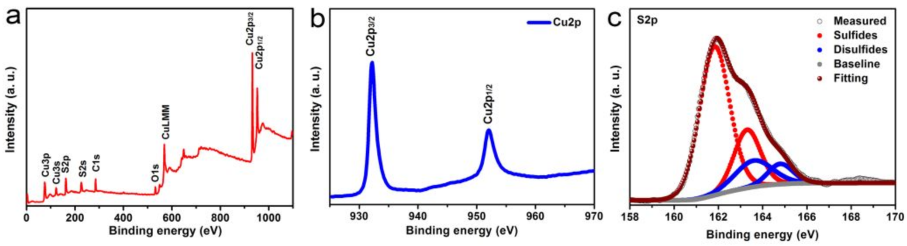

Figure S8. (a) XPS survey. High-resolution (b) Cu 2p and (c) S 2p spectra of CuS.

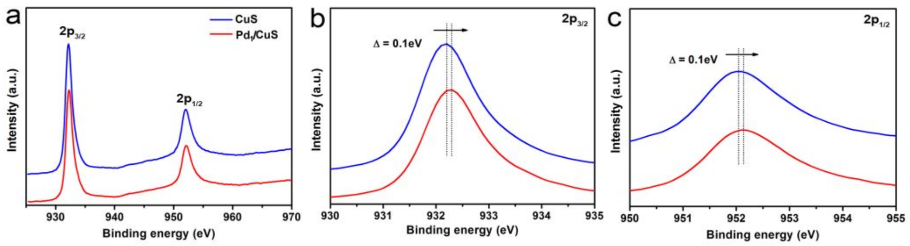

Figure S9. Comparison of Cu 2p spectra of CuS and Pd_1/CuS.

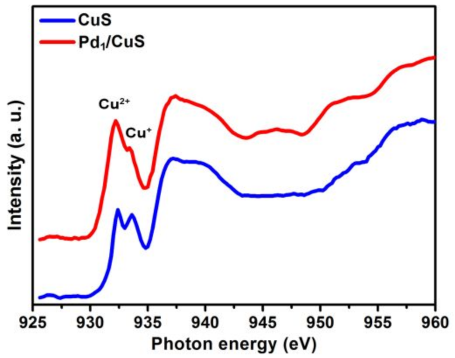

Figure S10. Cu L-edge XANES spectra of CuS and Pd_1/CuS.

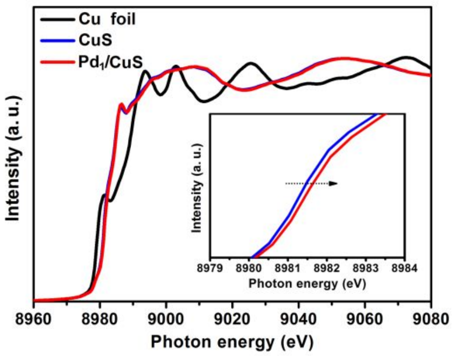

Figure S11. Cu K-edge XANES spectra of CuS and Pd_1/CuS.

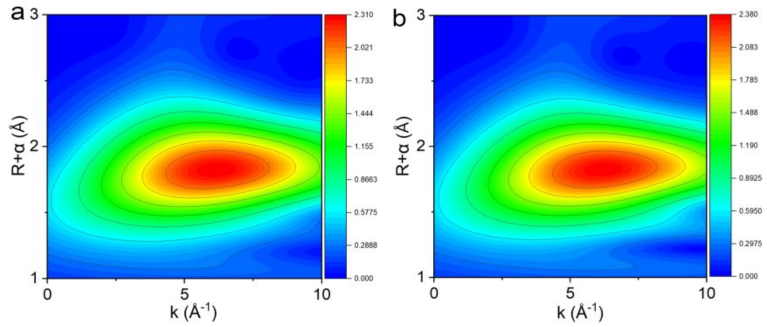

Figure S12. WT-EXAFS spectra of CuS and Pd_1/CuS at Cu K-edge.

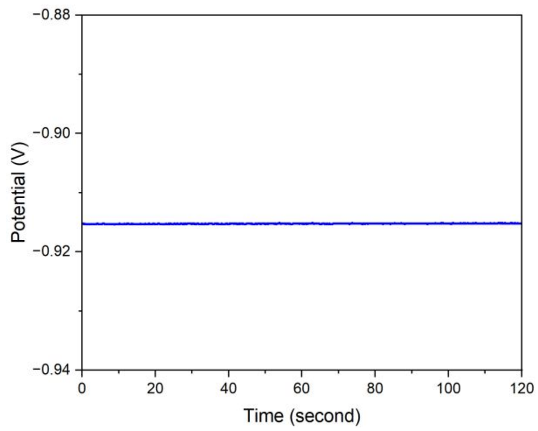

Figure S13. Reference Hg/HgO potential calibration using hydrogen reference electrode (PHY-RHE) in 1.0 M KOH containing 0.5 M KNO_3.

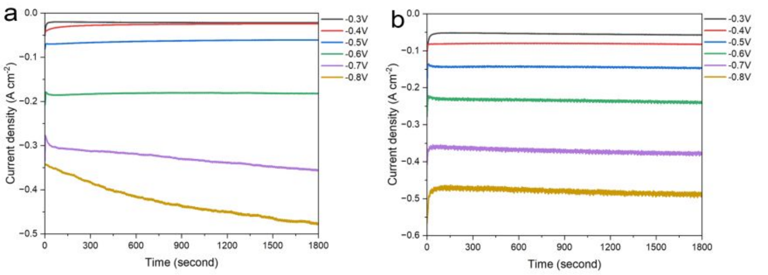

Figure S14. Chronoamperometry curves for (a) CuS and (b) Pd_1/CuS in 1.0 M KOH with 0.5 M KNO_3.

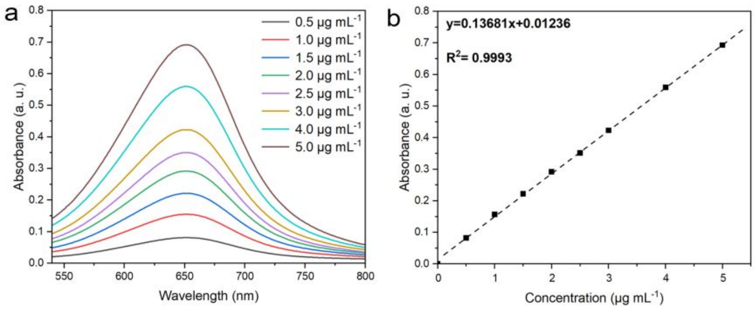

Figure S15. (a) UV-Vis absorption spectra and (b) calibration curve in different concentrations of NH_4Cl.

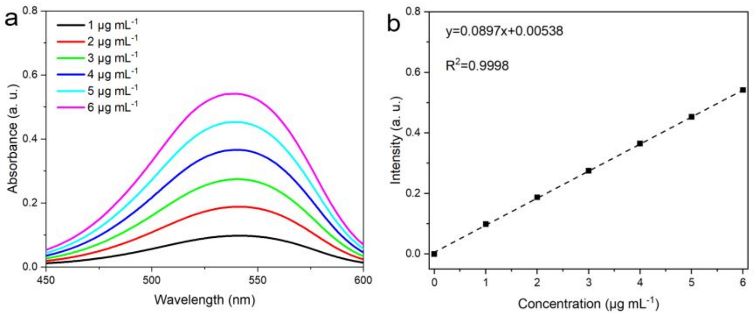

Figure S16. (a) UV-Vis absorption spectra and (b) calibration curve in different concentrations of KNO_2.

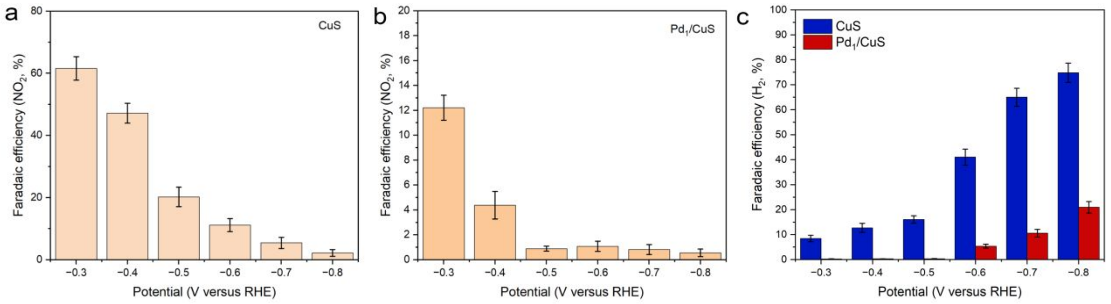

Figure S17. Faradaic efficiency for NO_2^- production on (a) CuS and (b) Pd_1/CuS catalysts across applied potentials. (c) Faradaic efficiency for H_2 production on CuS and Pd_1/CuS catalysts.

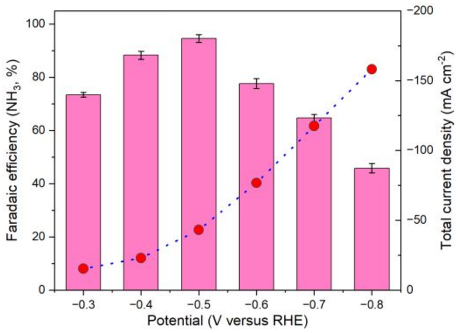

Figure S18. Faradaic efficiency and total current density of activated Pd_1/CuS in 1.0 M KOH containing 0.1M KNO_3.

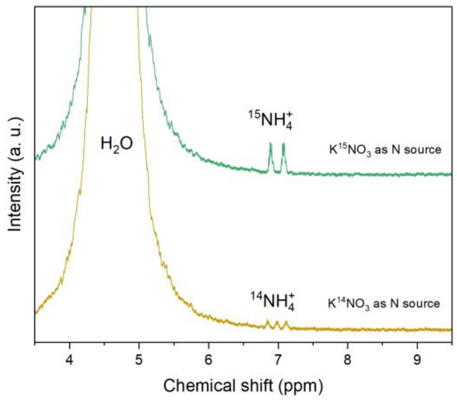

Figure S19. ^{1}H NMR data comparison with $^{15}NO_3^-$ (green) and $^{14}NO_3^-$ (yellow) as the reactant, respectively.

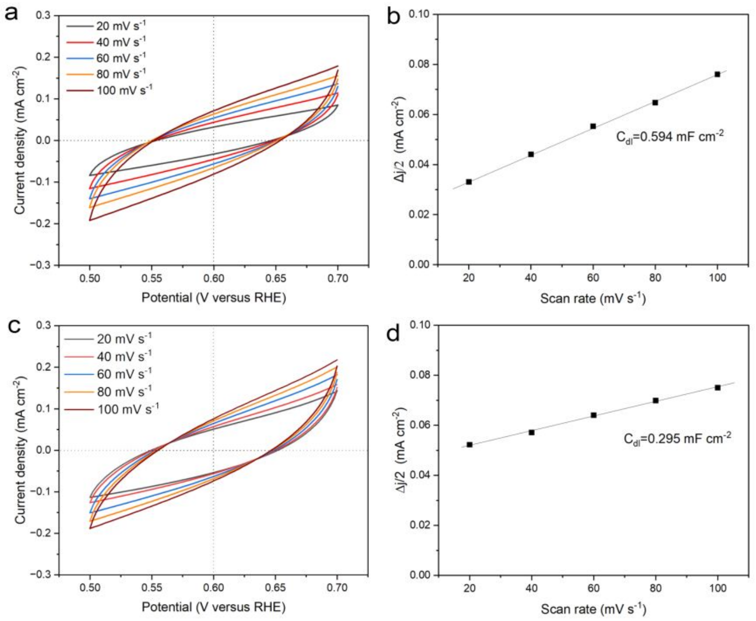

Figure S20. ECSA evaluation of (a, b) CuS and (c, d) Pd_1/CuS.

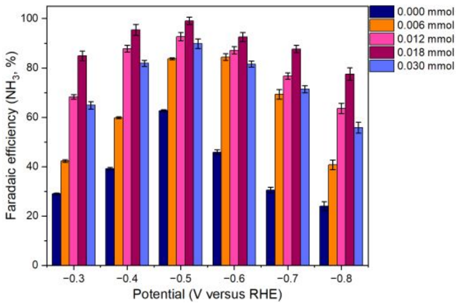

Figure S21. Faradaic efficiency comparison for ammonia production on CuS with different Pd usage across applied potentials.

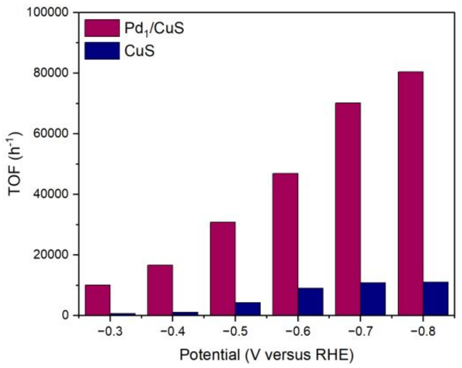

Figure S22. TOF values of both catalysts calculated from ECSA.

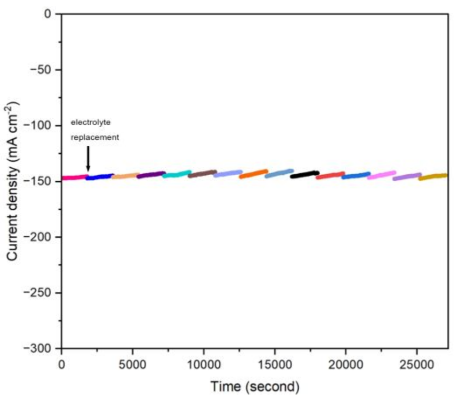

Figure S23. Cycling stability curves of Pd_1/CuS.

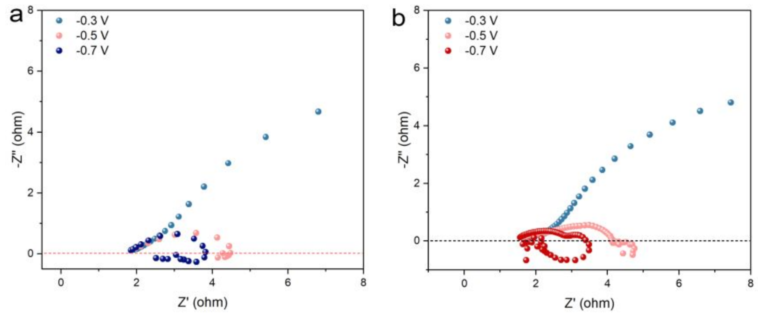

Figure S24. Nyquist plots of (a) Pd_1/CuS and (b) CuS.

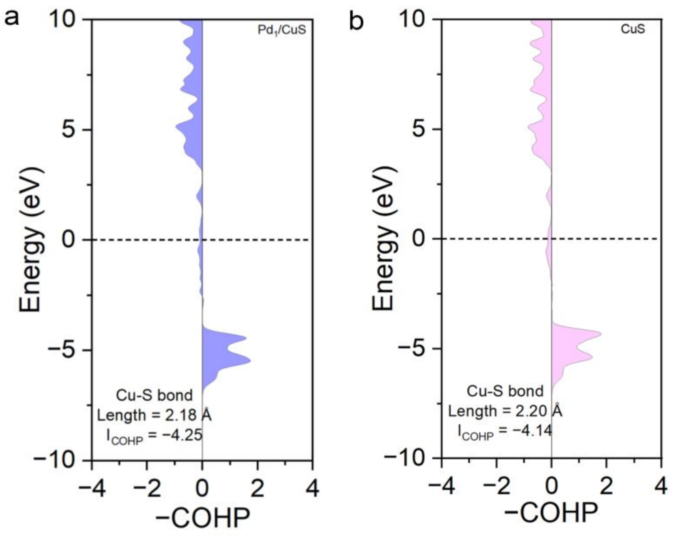

Figure S25. COHP analysis of Cu-S in (a) Pd_1/CuS and (b) CuS.

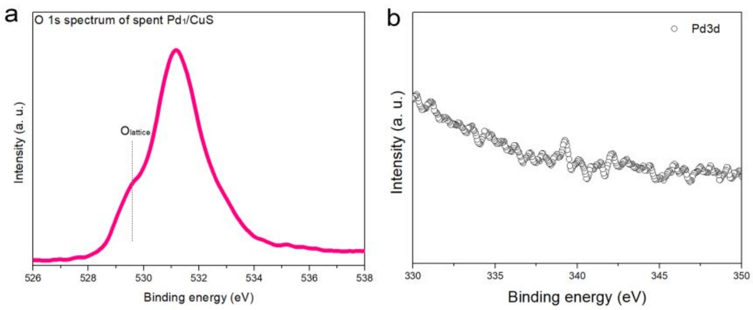

Figure S26. High-resolution XPS of (a) O 1s and (b) Pd 3d of post-reaction Pd_1/CuS.

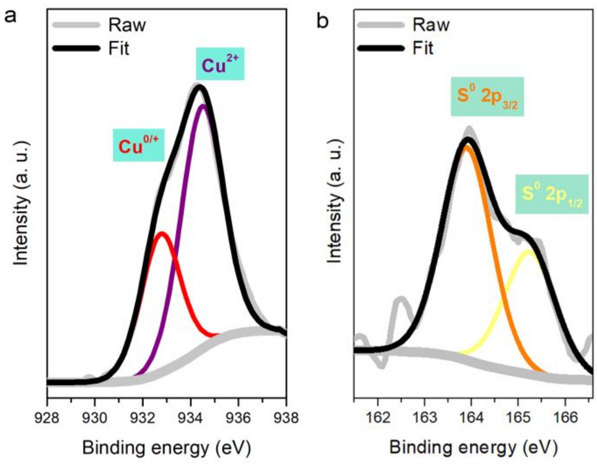

Figure S27. High-resolution XPS of (a) Cu 2p and (b) S 2p of post-reaction CuS.

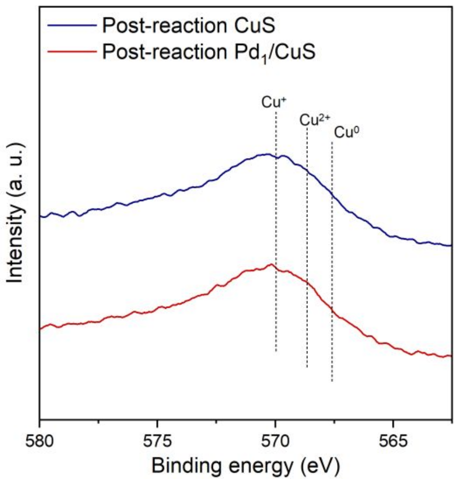

Figure S28. Cu LMM Auger spectra of CuS and Pd_1/CuS after reaction.

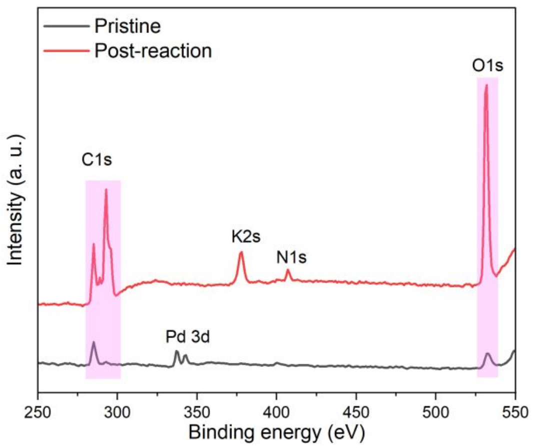

Figure S29. XPS survey spectra of pristine and post-reaction Pd_1/CuS.

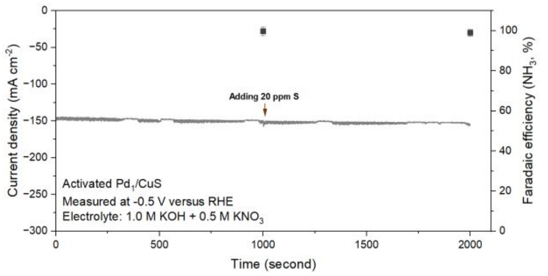

Figure S30. S addition experiments for Pd_1/CuS measured at -0.5 V versus RHE.

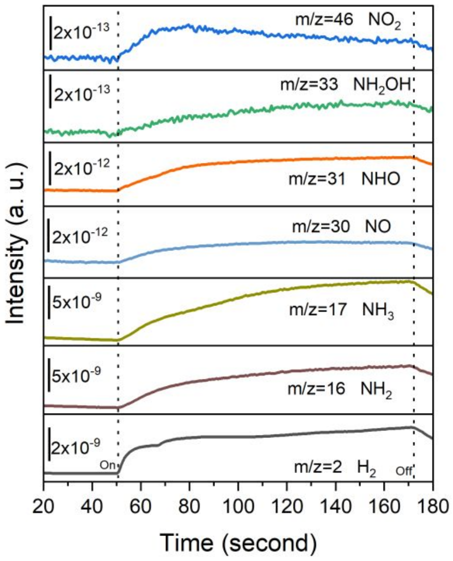

Figure S31. Online DEMS of electrochemically activated CuS.

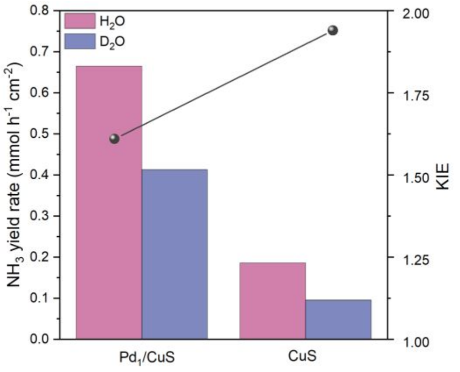

Figure S32. Calculated KIE values of Pd_1/CuS and CuS in the H_2O-based and D_2O-based electrolyte containing 1.0 M KOH with 0.5 M KNO_3.

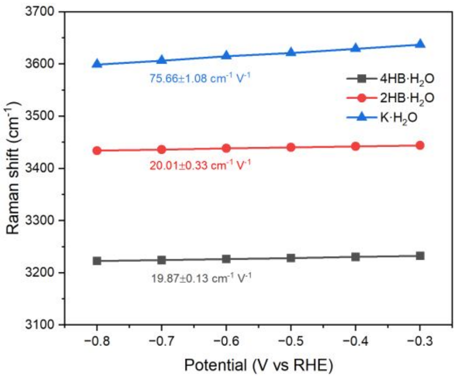

Figure S33. Potential-dependent Stark effect shift analysis of CuS from Figure 4f.

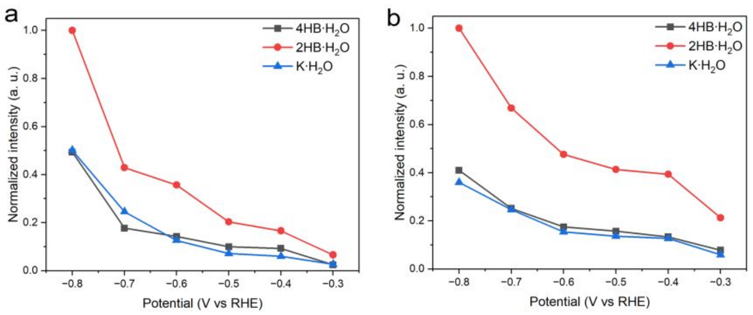

Figure S34. Comparison of normalized intensity of different interfacial water molecules for electrochemically activated (a) CuS and (b) Pd_1/CuS.

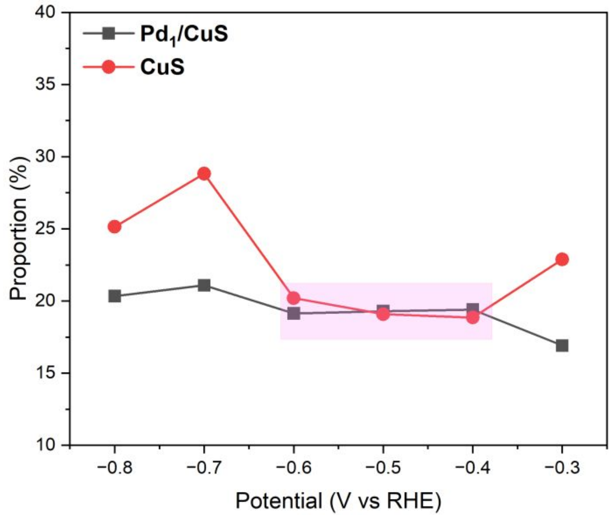

Figure S35. Proportion Comparison of K·H_2O at each potentials for activated Pd_1/CuS and CuS.

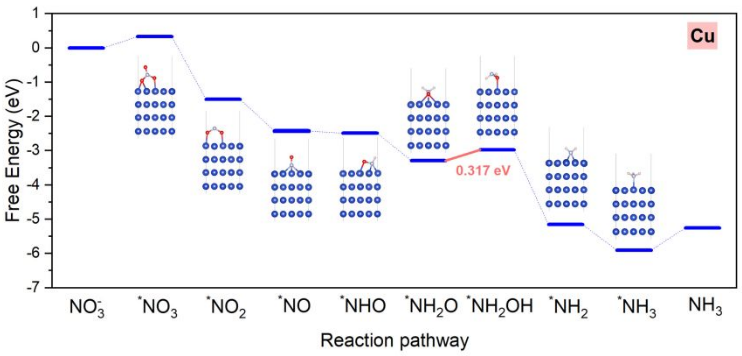

Figure S36. The reaction free energy profile of nitrate reduction on Cu catalyst.

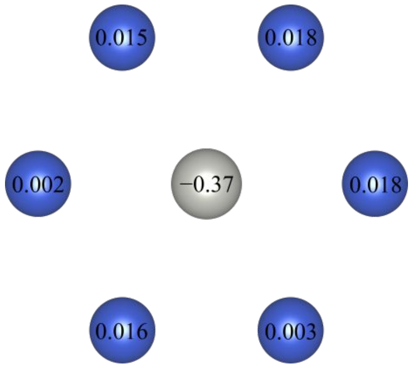

Figure S37. Bader charge analysis of Pd-doped Cu model.

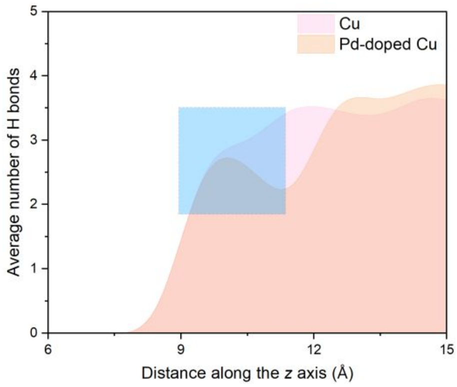

Figure S38. Qualitatively analyzed average hydrogen bond from AIMD simulations.

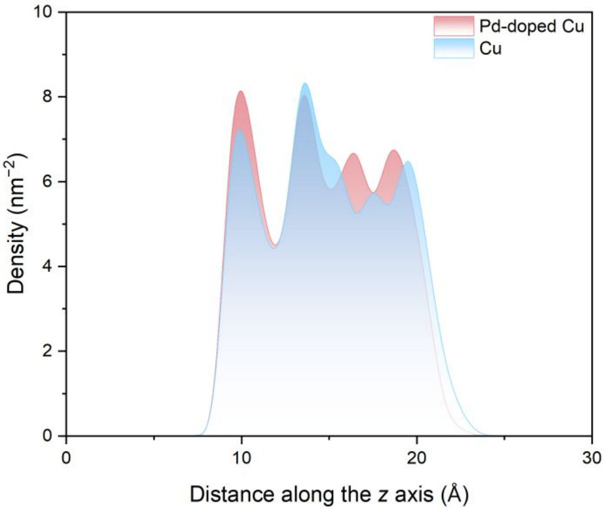

Figure S39. The density distribution of H atoms along the *z* axis on Cu and Pd doped Cu.

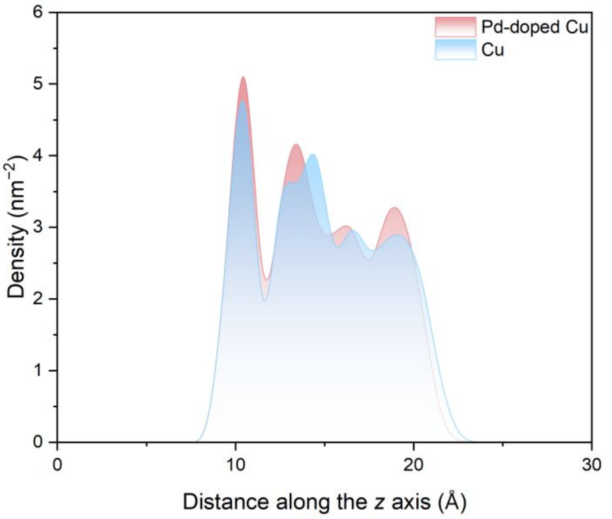

Figure S40. The density distribution of O atoms along the *z* axis on Cu and Pd doped Cu.

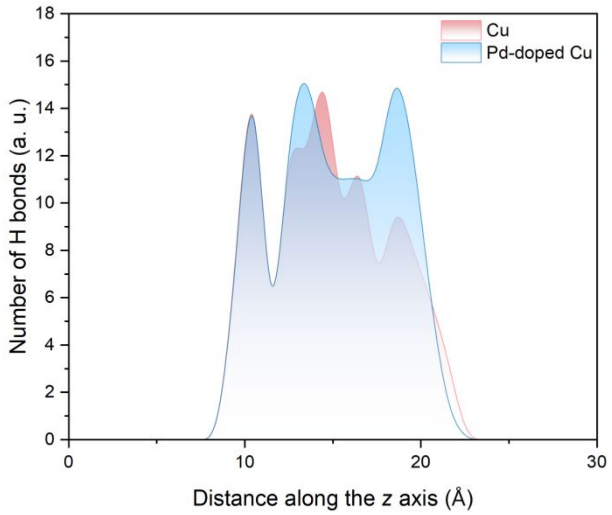

Figure S41. The number of hydrogen bonds along the *z* axis on Cu and Pd doped Cu.

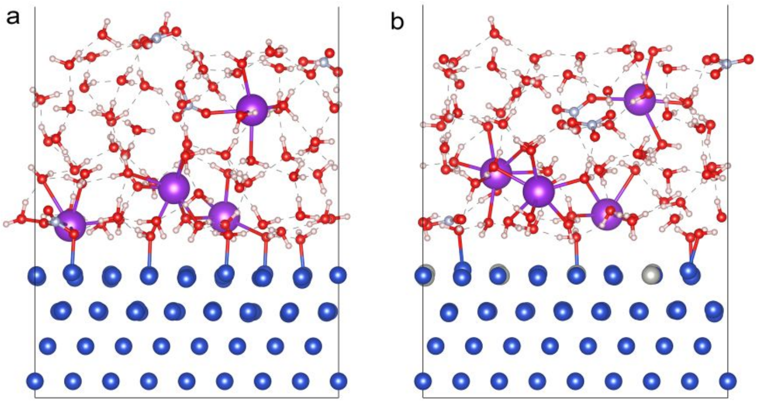

Figure S42. Snapshots of solvent/catalyst interface structures along the *z* direction for Cu and Pd doped Cu catalysts.

Table S1. ICP result of pristine and post-reaction Pd_1/CuS.

Sample	Element	Mass concentration (mg L^{-1})	Molar concentration (mmol L^{-1})	Molar ratio (%)
Pristine	Cu	27.758	0.4368	0.92
	Pd	0.421	0.0040	
Post-reaction (12h)	Cu	15.368	0.2418	0.50
	Pd	0.130	0.0012	
Post-reaction (24h)	Cu	18.346	0.2887	0.52
	Pd	0.162	0.0015	

Table S2. FT-EXAFS fitting data from Cu K-edge EXAFS.

Sample	Path[&1]	N[&2]	R [Å][&3]	$\lvert\Delta E_0\rvert$ [eV][&4]	σ^2 [10^{-3} $Å^2$][&5]	R-factor[&6]
CuS	Cu-S	2.97±0.25	2.27 (2)	9.17	4.59	0.010
Pd_1/CuS	Cu-S	3.02±0.23	2.27 (1)	9.12	4.99	0.005

[&1] Scattering paths

[&2] Coordination number

[&3] Bond length

[&4] Energy shift

[&5] Debye-Waller factor

[&6] Degree of curve coincidence

Table S3. FT-EXAFS fitting data from Pd K-edge EXAFS.

Sample	Path	N	R [Å]	$|\Delta E_0|$ [eV]	σ^2 [10^{-3} Å^2]	R-factor
PdS	Pd-S	4.00#	2.32(9)	1.77	5.81	0.018
Pd_1/CuS	Pd-S	3.72±0.54	2.33(1)	0.66	4.83	0.014

fixed parameter

Supplementary References

[1] Kresse, G.; Furthmüller, J. Efficient iterative schemes for ab initio total-energy calculations using a plane-wave basis set. *Phys. Rev. B* **1996**, *54*, 11169–11186.

[2] Kresse, G.; Furthmüller, J. Efficiency of ab-initio total energy calculations for metals and semiconductors using a plane-wave basis set. *Comput. Mater. Sci.* **1996**, *6*, 15–50.

[3] Kresse, G.; Joubert, D. From ultrasoft pseudopotentials to the projector augmented-wave method. *Phys. Rev. B* **1999**, *59*, 1758–1775.

[4] Perdew, J. P.; Burke, K.; Ernzerhof, M. Generalized gradient approximation made simple. *Phys. Rev. Lett.* **1996**, *77*, 3865–3868.

[5] Grimme, S.; Antony, J.; Ehrlich, S.; Krieg, H. A consistent and accurate ab initio parametrization of density functional dispersion correction (DFT-D) for the 94 elements H-Pu. *J. Chem. Phys.* **2010**, *132*, 154104.

[6] Momma, K.; Izumi, F. VESTA 3 for three-dimensional visualization of crystal, volumetric and morphology data. *J. Appl. Crystallogr.* **2011**, *44*, 1272–1276.

[7] Wang, V.; Xu, N.; Liu, J.-C.; Tang, G.; Geng, W.-T. VASPKIT: a user-friendly interface facilitating high-throughput computing and analysis using VASP code. *Comput. Phys. Commun.* **2021**, *267*, 108033.

[8] Henkelman, G.; Jónsson, H. Improved tangent estimate in the nudged elastic band method for finding minimum energy paths and saddle points. *J. Chem. Phys.* **2000**, *113*, 9978–9985.

[9] CP2K: An electronic structure and molecular dynamics software package - Quickstep: Efficient and accurate electronic structure calculations | J. Chem. Phys. | AIP Publishing.

[10] Humphrey, W.; Dalke, A.; Schulten, K. VMD: Visual molecular dynamics. *J. Mol. Graph.* **1996**, *14*, 33–38.
